# Venlafaxine-induced psychotic symptoms

**DOI:** 10.4103/0019-5545.58301

**Published:** 2009

**Authors:** A. T. Safeekh, Denzil Pinto

**Affiliations:** Department of Psychiatry, Father Muller Medical College, Mangalore - 575 002, India

**Keywords:** Psychotic symptoms, venlafaxine, social phobia

## Abstract

Venlafaxine, an antidepressant belongs to Serotonin Norepinephrine Reuptake Inhibitors (SNRI), blocks the synaptic reuptake of serotonin in lower doses and also blocks reuptake of norepinephrine in higher doses. In addition it also blocks dopamine reuptake in still higher doses. This last mechanism of action is found to cause psychotic symptoms. Very few cases are reported with this adverse effect of venlafaxine. Here is a case report where a 32 year-old male with social phobia developed delusions of persecution on two occasions when he was put on venlafaxine 150 mg/day, which responded to withdrawal of venlafaxine and a short course of antipsychotics. The delusions never reappeared when he was maintained on escitalopram.

## INTRODUCTION

A specific pharmacological agent is chosen depending primarily on the target symptoms, its mode of action, and the profile of its side effects. The interaction and intermodulation of various neurotransmitters in the brain are so complex and often the iatrogenic symptoms complicate the clinical picture. When multiple drugs or drugs with multiple modes of action are used the complications become more unpredictable. Venlafaxine a SNRI, (serotonin and norepinephrine reuptake inhibitor), which inhibits 5HT reuptake in lower doses and inhibits norepinephrine (NE) reuptake in higher doses, is a very potent antidepressant. Apart from inhibiting serotonin and norepinephrine reuptake it also inhibits dopamine reuptake in very high doses.[1] A case of erotomania in a patient with major depressive disorder when she was put on 225-300 mg/day of venlafaxine was reported by Admou and Hale.[2] Here we report a case where a patient with social phobia developed delusions of persecution when he was treated with venlafaxine 150 mg/day.

## CASE REPORT

A 32-year unmarried male who is working as a high school teacher presented with difficulty in conversing with women, choking sensation and flushing of face while taking class, and being unable to speak in the parent-teacher association meetings, since two years after working as a teacher. He often used to stop the class in between and also used to avoid social functions because of the above mentioned symptoms. He was always worried that people would make fun of his lack of social skills and his incapabilities. His biological functions were not disturbed. Patient was using alprazolam 0.5 mg every morning, which he found to be helpful in the reduction of severity of symptoms. There was no history of any substance use. There was no contributory past history or family history. Premorbidly he was fairly well adjusted. His physical examination was within normal limits. No depressive cognitions, obsessions, delusions or perceptual disturbances were found in the mental status examination (MSE) and his cognitive functions were intact. A diagnosis of social phobia was made (F 40.1; ICD 10). Investigations including complete blood count, blood sugar, and thyroid functions were within normal limits. Treatment options were discussed with the patient. As he was residing far away from the hospital he would not be able to avail leave quite often to attend the sessions and behavior therapy was ruled out. He was started on escitalopram 10 mg and clonazepam 0.5 mg in the morning and advised to stop using alprazolam. Although he was asked to report after two weeks, he came one week later complaining of abdominal discomfort and nausea. Escitalopram was stopped and venlafaxine 75 mg/ day was started. He came for follow up after one month with marginal reduction in the severity of symptoms and venlafaxine was increased to 150 mg. On the next follow up after one month there was significant improvement in the symptoms and the same medication was prescribed. The patient reported two weeks later saying that he was so tensed as two of his colleagues were trying to harass him. A detailed interview revealed that he had developed a persecutory delusion that those two colleagues wanted to expel him from the school and they were sending spies to follow him. He also reported that they were making good looking girls stand next to him in the bus so that he would be tempted to molest them. Venlafaxine was stopped and olanzapine 5 mg at night was prescribed. He was advised to report after one week with his sister. On the next follow up, the patient reported with his sister as advised. As the patient had told his sister about his colleagues' harassment she had already enquired about it through a friend of her's who was working in the same school and found that to be untrue. The patient's sister reported that on the way to the hospital, in the train, he appeared very fearful and pointed out two men sitting in the same compartment and complained to her that they were sent by his colleagues to follow him, to find out where he was going, and later they were going to spread rumors that he was mad, as he was visiting a psychiatrist. Olanzapine was increased to 10 mg and during his next follow up after one month his delusions had disappeared. Olanzapine was reduced to 5 mg and venlafaxine 75 mg/day was restarted. During the next few follow ups, Olanzapine was gradually stopped over a period of two months, while venlafaxine 150 mg/day was continued. The patient reported two months later with the same delusions of persecution and reference. Olanzapine 10 mg was reintroduced and venlafaxine was stopped. As the delusions disappeared. Olanzapine was tapered once again and stopped within two months. As his social phobic symptoms were persisting escitalopram 10 mg was started. Although the patient had reported gastrointestinal adverse effects when he was prescribed escitlopram previously, he did not develop any gastrointestinal or other adverse effects this time when escitalopram was reintroduced. During no time of his illness were elated or irritable mood or any other manic symptoms reported or observed. During the entire course of treatment clonazepam 0.5 mg was continued. The patient is maintaining well on escitalopram 10 mg and clonazepam 0.5 mg for the last eighteen months. He got married one year ago.

## DISCUSSION

Emergence of delusions during the course of social phobia made the authors reconsider the validity of their initial diagnosis. However, factors such as the absence of a past history, family history or substance use, the fast remission of delusions with a short course of antipsychotic and re-emergence of symptoms on reintroduction of venlafaxine pointed to the relationship between venlafaxine and psychotic symptoms. Moreover, the patient did not develop any psychotic symptoms after venlafaxine was stopped, and he was maintained on escitalopram during the last eighteen months.

The dopamine reuptake inhibiting action of venlafaxine in high doses is described by Stahl,[1] which might be the possible reason for venlafaxine-induced psychotic symptoms.

Admou and Hale[2] had reported a case where a 39 year-old lady with major depressive disorder developed delusions of love on two separate occasions, on treatment with venlafaxine in the doses of 225-300 mg/day. Each time the delusions remitted after lowering the dose of venlafaxine to 75-150 mg/day.

Propafenone is found to cause increased serum concentration of venlafaxine. A case of organic psychosis during the treatment of a patient with Bipolar disorder has been reported by Pfeffer and Grube, due to an increase in serum concentration of venlafaxine on account of propafenone.[3] Anghelescu *et al*.,[4] have reported visual hallucination following venalafaxine treatment in a lady with posterior cerebral artery infarction. Visual hallucinations have been reported during the treatment with other antidepressants including tricyclics and specific serotonin inhibitors also.[5] Theoretically the dopamine reuptake inhibition of venlafaxine should occur only in high doses. However, the present case shows that even in a moderate dose it might produce dopamine reuptake inhibition and hence demonstrate the need to monitor patients on venlafaxine, for the emergence of psychotic symptoms.
